# Methotrexate Toxicity-Induced Pancytopenia and Mucocutaneous Ulcerations in Psoriasis

**DOI:** 10.7759/cureus.66222

**Published:** 2024-08-05

**Authors:** Devipriya Surapaneni, Sharath Chandra Dasi, Noel Sam, Jagadeesan M

**Affiliations:** 1 Internal Medicine, Saveetha Institute of Medical and Technical Sciences, Chennai, IND; 2 General Medicine, Saveetha Institute of Medical and Technical Sciences, Chennai, IND

**Keywords:** folinic acid rescue, methotrexate-induced pancytopenia, chronic kidney disease (ckd), plaque psoriasis, methotrexate toxicity

## Abstract

Methotrexate (MTX) is a commonly used immunosuppressant and chemotherapeutic agent, widely prescribed for autoimmune diseases such as psoriasis, rheumatoid arthritis, and certain malignancies. It functions by inhibiting dihydrofolate reductase, leading to impaired DNA synthesis and cell proliferation. While generally well-tolerated, MTX has a narrow therapeutic index, and its adverse effects can be severe, including hepatotoxicity, pulmonary toxicity, and hematological complications such as pancytopenia. Pancytopenia involves the reduction of all three blood cell lines and can result in significant morbidity and mortality. The risk of MTX toxicity is notably higher in patients with renal impairment, as the kidneys are the primary route of drug excretion. Renal dysfunction can lead to the accumulation of MTX, enhancing its toxicity. Numerous studies and case reports have highlighted the risks of MTX toxicity, especially in patients with renal impairment. Pancytopenia can present insidiously, with symptoms such as mucosal ulcers, fever, and generalized weakness, making early detection crucial.

We report a case of a male patient in his late 40s with a complex medical history, including psoriasis, insulin-dependent type 2 diabetes mellitus, chronic kidney disease (CKD) stage 3b, and coronary artery disease (CAD). The patient presented to the emergency department with a one-week history of fever, generalized weakness, mouth sores, and a five-day history of bilateral lower limb swelling and pain. Vital signs were stable, but physical examination revealed pallor, large ulcerative lesions in the buccal mucosa, and erythematous, scaly lesions on the lower limbs. The patient's medication history included methotrexate, which he had stopped two months prior but was inadvertently resumed at an increased dose two weeks prior to presentation. Laboratory findings revealed pancytopenia with worsening trends, prompting a bone marrow biopsy that showed hypocellular marrow. The patient's CKD likely exacerbated the MTX toxicity due to impaired drug clearance, leading to pancytopenia. Treatment included intravenous leucovorin, blood and platelet transfusions, and granulocyte-macrophage colony-stimulating factor (GM-CSF). Despite initial critical presentation, the patient showed significant improvement, with recovery of blood counts and resolution of symptoms. He was discharged with stable hemoglobin, platelet, and white blood cell counts.

## Introduction

Psoriasis is a frequently seen inflammatory, proliferative immune-mediated skin disorder. It is identified by lasting scaly pink patches found on the elbows, knees, and scalp [1]. It is characterized by hyperproliferation and abnormal differentiation of epidermal keratinocytes, lymphocyte infiltration consisting mostly of T lymphocytes, and various endothelial vascular changes in the dermal layer [2].

Methotrexate (MTX) is used worldwide for the treatment of moderate to severe plaque psoriasis, psoriatic erythroderma, generalized pustular psoriasis, nail psoriasis, palmoplantar psoriasis, and psoriatic arthritis [3]. Methotrexate works by competitively inhibiting dihydrofolate reductase, which leads to a reduction in the production of folate cofactors required for nucleic acid synthesis. Its use for the treatment of the above conditions was approved by the United States Food and Drug Administration in 1972.

Methotrexate, although effective in the majority of patients, has the potential for hepatotoxicity and is contraindicated in the following settings: pregnancy, individuals with renal impairment, hepatitis, or cirrhosis, alcoholics, and patients with leukemia or thrombocytopenia [4]. Common indicators of methotrexate toxicity are bone marrow suppression and the development of oral and gastrointestinal ulcers. A less frequent sign of methotrexate toxicity is the painful erosion of psoriatic plaques, and its diagnosis is associated with a fear of impending pancytopenia [5].

Methotrexate is primarily eliminated by the kidneys, with 80%-90% excreted unchanged in the urine. To avoid toxic effects, prescribing guidelines recommend that methotrexate be started at a low dose in patients with chronic kidney disease (CKD) [6]. Our patient, a known case of chronic kidney disease, has an increased predisposition to developing methotrexate toxicity and its potential side effects. Although physicians usually start a low-dose regimen in the treatment of psoriasis and other conditions such as rheumatoid arthritis, it can be potentially toxic in the background of chronic kidney disease, especially in patients with concomitant comorbidities.

We present a case of a male patient in his late 40s with a past medical history of psoriasis, chronic kidney disease (CKD) stage 3b, and other comorbidities, who was admitted with signs of systemic infection, painful oral ulcers, and bilateral lower limb swelling. After a comprehensive evaluation, a provisional diagnosis of MTX-induced pancytopenia was established following the identification of inappropriate medication dosing amid worsening renal function. The patient's pancytopenia was managed with IV leucovorin, blood transfusions, and granulocyte-macrophage colony-stimulating factor (GM-CSF), resulting in clinical improvement and resolution of cytopenias.

This case highlights the importance of careful medication management in patients receiving MTX, particularly those with renal impairment, and the need for vigilance regarding potential toxicities.

## Case presentation

**Figure 1:**
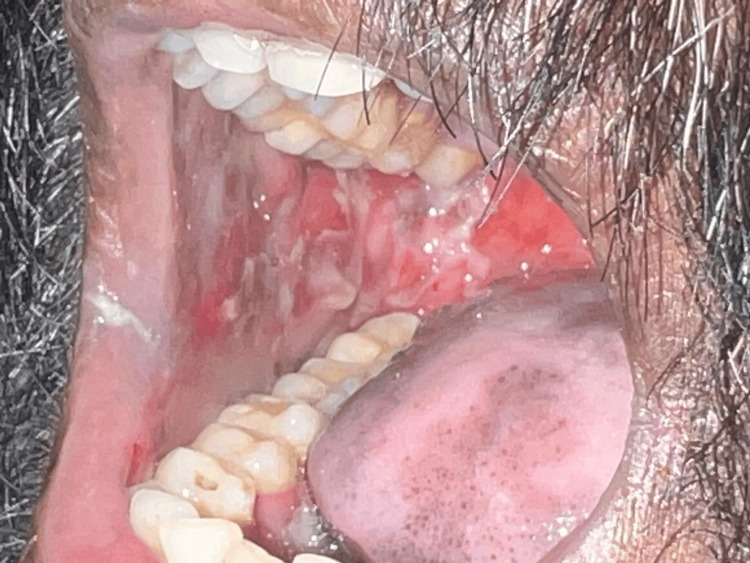
Oral mucocutaneous ulcerations Multiple cutaneous ulcerations and erosions in confined to the buccal mucosa with mucosal erythema

A male patient in his late 40s with a past medical history of psoriasis, insulin-dependent type 2 diabetes mellitus, chronic kidney disease stage 3b, and coronary artery disease presented to our emergency department with chief complaints of fever since past one week, generalized weakness, mouth sores since past one week, and bilateral lower limb swelling and pain since past five days. Vitals on presentation were blood pressure of 140/80 mmHg, pulse rate of 84/minute, respiratory rate of 18 breaths/minute, temperature of 99.4°F, and saturation of 99% under room rate. Physical examination revealed pallor and large ulcerative lesions in the buccal mucosa (Figure 1), which were reported to be painful.

**Figure 2:**
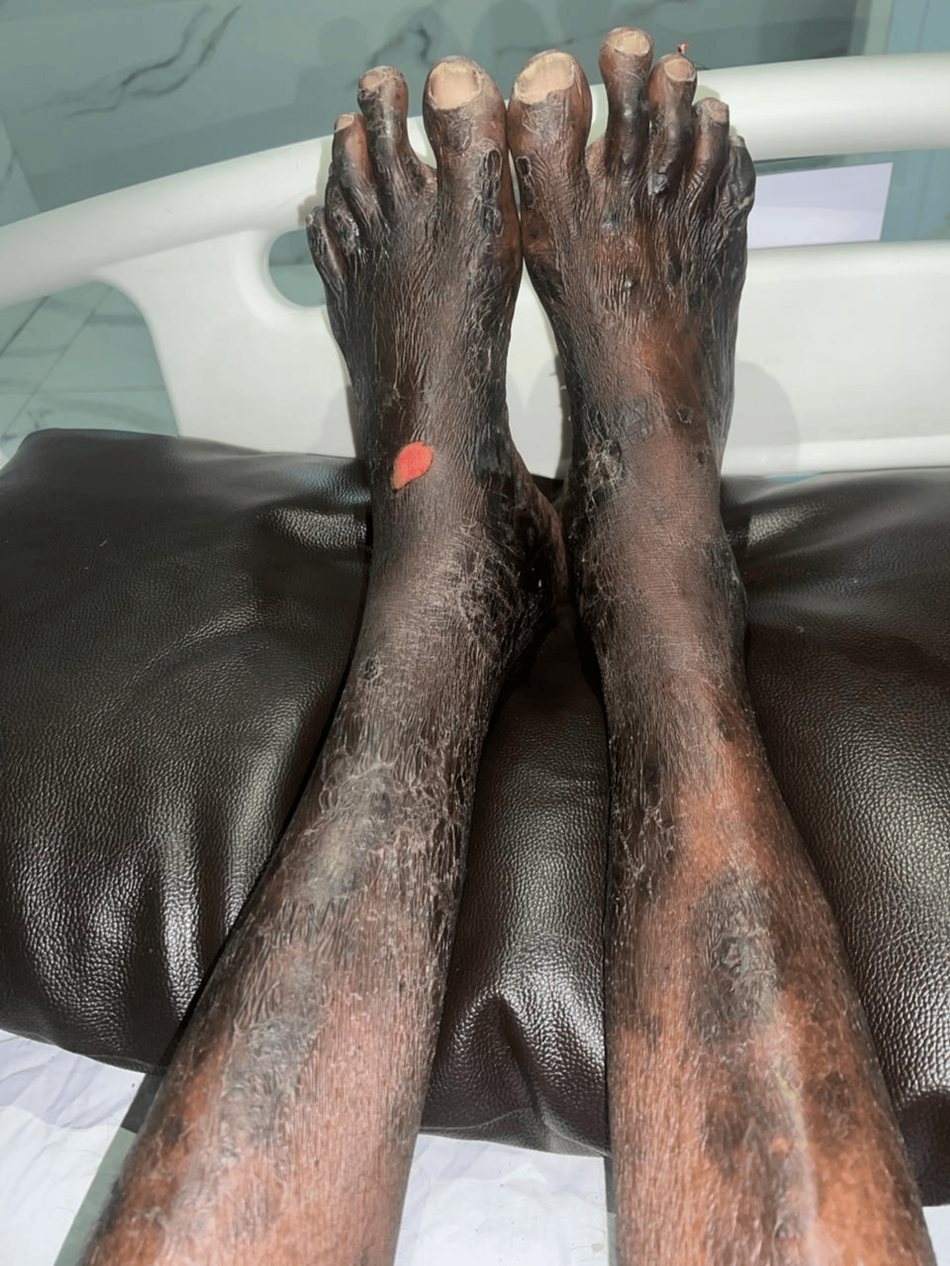
Ulcerations in plaque psoriasis Necrosis and ulceration of plaque psoriasis in the bilateral lower limb with a superficial ulcer of size 2.5 × 2 cm seen on the dorsal aspect of the right foot

Bilateral lower limb swelling, significant warmth and tenderness, and sharply demarcated, hyperpigmented, scaly, erythematous, and ulcerative lesions were noted on the extensor surface of both lower limbs (Figure 2).

**Figure 3:**
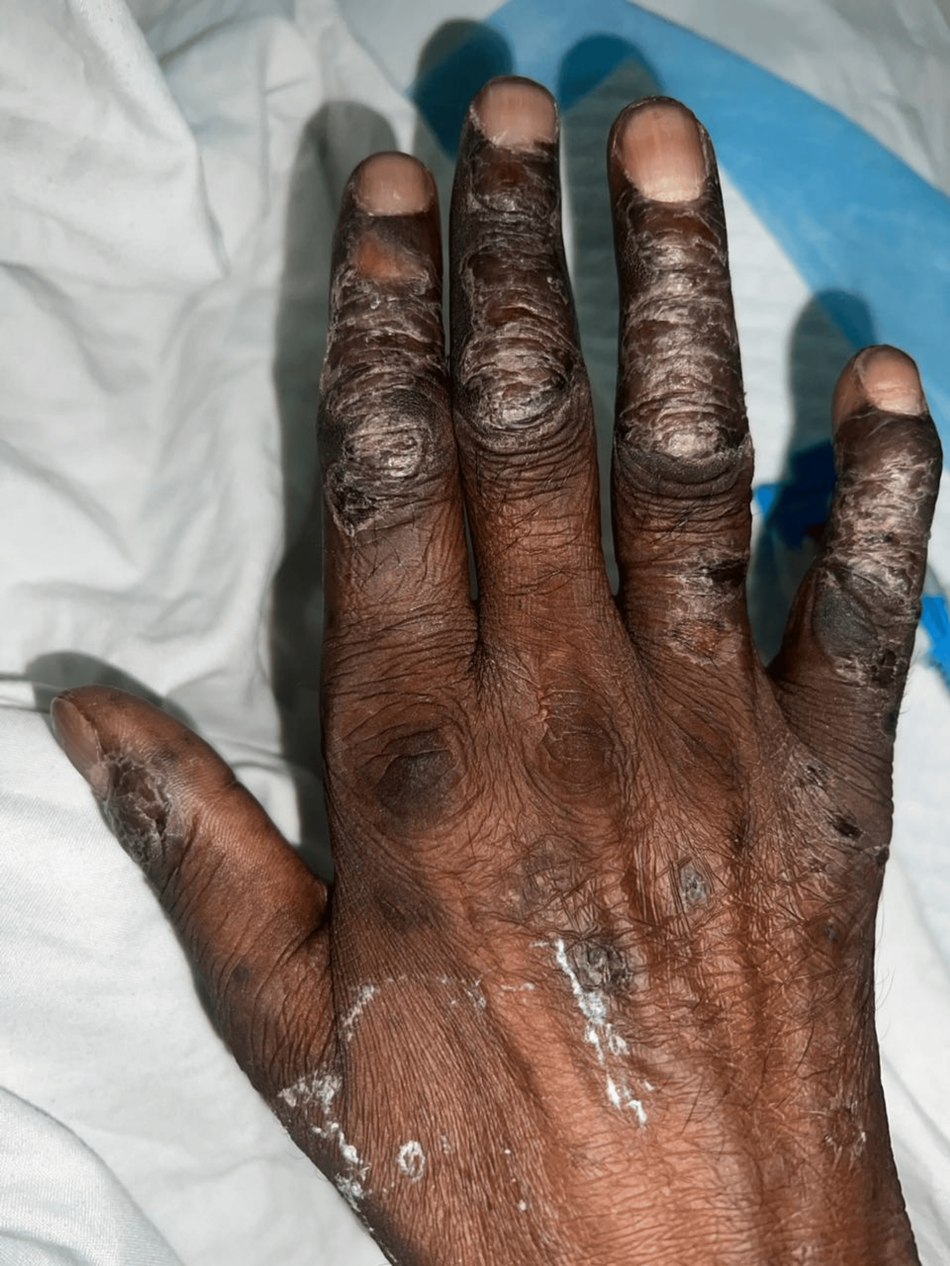
Plaque psoriasis Plaque psoriasis in the bilateral upper limb with erosion and silver scaly lesions seen on the extensor aspect of the hand

A few silver scaly lesions were also noted on the dorsal aspect of the fingers of both upper limbs (Figure 3).

The rest of the physical and systemic examination was normal. The patient is not a known smoker or alcoholic. He gives a drug history of tablet methotrexate 5 mg once weekly for the past 10 years for psoriasis. The patient has a history of coronary artery disease (CAD) and heart failure with reduced ejection fraction, and he is on regular treatment, although the details of the treatment are not available. He was admitted to our hospital two months ago in the cardiology department. During that admission, he was diagnosed with an anterior wall myocardial infarction (AWMI) and managed conservatively. A coronary angiogram revealed double vessel disease. Additionally, during that time, he was diagnosed with cardiorenal syndrome (CRS), with a creatinine level of 2 mg/dL. Methotrexate (MTX) was withheld as an angiogram and other procedures were being performed, and his condition needed to be stabilized.

During this admission, his laboratory investigations revealed deranged renal parameters, with a urea level of 53 mg/dL and a creatinine level of 2.2 mg/dL. His eGFR was calculated to be 31.5 mL/minute/1.73 m², and he received appropriate management for cardiorenal syndrome type 2 (CRS type 2). Dermatology, cardiology, and nephrology departments were consulted, and appropriate treatment was initiated. The patient was initially diagnosed with bilateral lower limb cellulitis and uncontrolled type 2 diabetes mellitus and was managed with cefoperazone/sulbactam, clindamycin, antiplatelets, diuretics, beta-blockers, topical steroids for psoriasis, and other supportive measures.

On the third day of hospital admission, his laboratory parameters revealed pancytopenia, which showed a persistent decreasing trend. On day 4 and day 5, in view of suspected neutropenic sepsis, the patient was shifted to the ICU and was started on neutropenic precautions. Antibiotics were escalated to meropenem, and the patient was managed with multiple blood and platelet transfusions and granulocyte-macrophage colony-stimulating factor for pancytopenia.

**Figure 4:**
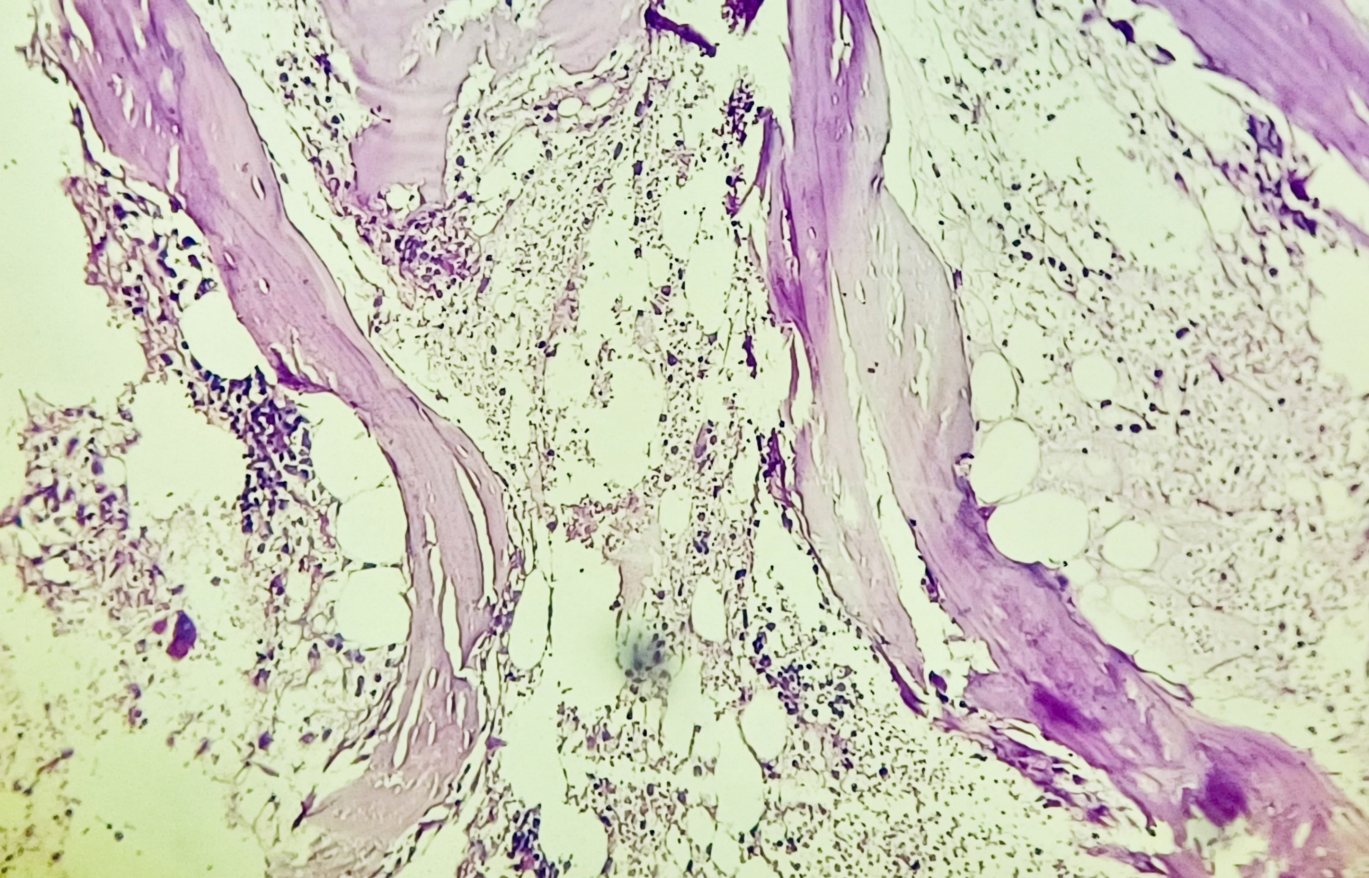
Bone marrow biopsy showing hypocellular marrow

Blood cultures after five days of incubation showed no growth. Urine culture and wound culture showed no growth. Despite the above treatment, pancytopenia was worsening, and there was no improvement in the patient's general condition, which made us think of an alternative diagnosis. A bone marrow biopsy was done, which showed hypocellular bone marrow with normoblastic erythroid hyperplasia (Figure 4).

Careful medication reconciliation revealed that the patient had taken two doses of tablet methotrexate 10 mg once weekly for two weeks prior to admission, and there was no dose adjustment done after he was diagnosed with CRS type 2. The onset and worsening of pancytopenia and mucosal ulcers correlated to the timeline of starting the medications. The impaired renal function might have additionally contributed to MTX toxicity, as the kidney is the primary route of excretion of the drug. MTX levels at the time of admission were not available. A provisional diagnosis of MTX-induced pancytopenia was made, and the patient was managed with intravenous leucovorin 10 mg/m^2^ every six hours; a total of 10 doses were given. Throughout the course of the ICU stay, the patient received daily blood transfusions, platelet transfusions, and subcutaneous GM-CSF. The targeted hemoglobin level was 7.5 g/dL, and platelet levels were 20,000/mm^3^, considering there were no bleeding manifestations. A significant improvement in pancytopenia was noticed from day 9 and showed a consistent increasing trend. The patient's general condition, oral mucosal ulcers, and skin lesions improved considerably, and he completed his antibiotic course. There was a remarkable improvement in his laboratory parameters, and the patient was discharged with a hemoglobin of 8.2 g/dL, a platelet count of 120,000/mm^3^, and a white blood cell count of 8,400/mm^3^ (Table 1).

**Table 1:** Laboratory investigations on days 1, 3, 4, 5, 8, 9, and 12 of admission RBC: red blood cell, PCV: packed cell volume, MCV: mean corpuscular volume, MCH: mean corpuscular hemoglobin, MCHC: mean corpuscular hemoglobin concentration, RDW: red cell distribution width, TLC: total leucocyte count, ANC: absolute neutrophil count

Laboratory parameters	Day 1	Day 3	Day 4	Day 5	Day 8	Day 9	Day 12	Reference range
Hemoglobin (g/dL)	11.9	5.2	6.5	9.7	7.4	7.2	8.2	12-15
Total RBC ( mill/mm^3^)	4.37	1.89	2.37	3.55	2.73	2.64	2.75	4.5-5.5
PCV (%)	36.8	16.3	20.1	29.6	22.5	21.8	28.2	40-50
MCV (fl)	84.2	86.2	84.8	83.4	82.4	82.6	84.1	83-101
MCH (pg)	27.2	27.5	27.4	27.3	27.1	27.3	29.2	27-32
MCHC (g/dL)	32.3	31.9	32.3	32.8	32.9	33	33.9	31.5-34.5
RDW (%)	13	14.8	14.6	14.5	14.5	14.6	13.8	11.6-14
Platelet count (lakhs/mm^3^)	1.89	0.80	0.42	0.21	0.12	0.55	1.2	1.5-4.5
TLC ( cells/mm3)	5,800	1,630	1,050	880	1,300	2,660	8,400	4,000-10,000
Neutrophils (%)	69.6	71.8	61.9	80.7	13.8	43.2	58.9	40-80
Lymphocytes (%)	21	22.7	30.5	17	43.1	27.1	17	20-40
Monocytes (%)	5.3	0	0	0	13.1	15	5.2	2-10
Eosinophils (%)	3.6	4.9	7.6	2.3	30	14.7	3.5	1-6
Basophils (%)	0.5	0.6	0	0	0	0.	0.4	<1-2
ANC (%)	4,030	1,170	650	710	180	1,150	6,090	2,000-7,000

## Discussion

MTX is a folate antagonist. It is used in the treatment of various autoimmune conditions, malignancies, etc. Its use has been accepted worldwide because of its good clinical response and comparatively fewer side effects. Understanding the spectrum of low-dose methotrexate-induced adverse events is as important as understanding its therapeutic benefits. Our knowledge of the nature and mechanisms of adverse events caused by methotrexate in patients is continually growing.

As noted by Day et al. [7], adverse drug effects may be classified as type A (dose-dependent (e.g., MTX gastrointestinal toxicity)), type B (idiosyncratic (e.g., MTX pneumonitis)), type C (resulting from long-term therapy but anticipated, based on overall drug exposure (e.g., MTX hepatotoxicity)), and type D (delayed effects occurring even after discontinuation of the drug (e.g., MTX in the first trimester of pregnancy, inducing teratogenesis)).

Potential severe adverse effects are more focused on hepatotoxicity, pulmonary toxicity, and risk of infection. Hematological reactions are less frequently encountered. Myelosuppression and pancytopenia are the most common hematological toxicities that usually occur later during low-dose MTX treatment, the incidence of which has been estimated to be 2%-4%. Pancytopenia due to MTX toxicity is unpredictable and underreported.

The potential risk factors for methotrexate toxicity were acute renal failure, hypoalbuminemia, and concomitant use of proton pump inhibitors (PPIs) and non-steroidal anti-inflammatory drugs (NSAIDs). It is contraindicated in patients with eGFR < 30 mL/minute [7]. Folic acid supplementation has shown proven benefits in reducing the incidence of liver impairment, but evidence of protective effects on hematological derangements is still unknown. The primary route of elimination of MTX is through the kidneys. Hence, these fatal complications can occur in patients with impaired renal function and should be closely monitored. Methotrexate toxicity leading to pancytopenia is a serious adverse effect that requires prompt intervention.

Pancytopenia as a rare side effect of low-dose MTX therapy was reported as early as 1996 in a meta-analysis conducted by Gutierrez-Ureña et al. [8]. A literature review was conducted to study the incidence of pancytopenia due to low-dose MTX therapy in rheumatoid arthritis spanning over the prior 15 years. An overall incidence of 1.4% was observed.

A study conducted by Lim et al. [9] at Norwich revealed that 25 patients developed MTX-induced pancytopenia. The median dose was 12.5 mg once weekly, with a median duration of treatment of three years. Pancytopenia severity correlated with the dose (P = 0.04). Risk factors included renal insufficiency, pre-existing folate deficiency, elderly population, and hypoalbuminemia. This was the largest reported individual case series of MTX-induced pancytopenia.

Leucovorin, also known as folinic acid, is used as a rescue agent in cases of methotrexate (MTX) toxicity. In cases of MTX toxicity, leucovorin is administered to "rescue" normal cells from the toxic effects of methotrexate by providing a source of reduced folate, facilitating the recovery of affected cells. Leucovorin is most effective when started within 24 hours of methotrexate administration. In our case, there was a delay in diagnosis due to incomplete drug history, resulting in a later initiation of leucovorin. Despite this delay, the administration of leucovorin remains beneficial for the patient. Off-label uses include neoadjuvant treatment in bladder cancer, as a cofactor in methanol toxicity, in the treatment of advanced esophageal cancer, advanced gastric cancer, advanced pancreatic cancer, prevention of hematological toxicity of pyrimethamine in patients with AIDS, and the treatment of ectopic pregnancy (along with methotrexate) [10].

A multicenter retrospective study done by Singh et al. [11] in which 21 patients with psoriasis with MTX toxicity were included showed 20 patients having mucocutaneous ulcerations and hematological abnormalities. All cases were treated with folinic acid, and 85% recovered.

Leucovorin is used in the management of such patients as it was in our patient. With the administration of intravenous leucovorin, the patient's laboratory parameters and general condition showed a dramatic improvement. Serum MTX levels before and after treatment are ideally necessary but could not be taken in our patient due to logistic reasons and lack of resources. The dose should be titrated accordingly in ideal situations. Studies have shown that GM-CSF and steroids for MTX-induced myelosuppression are useful. Frequent laboratory monitoring and careful dose increments are the cornerstones of early diagnosis and prevention of MTX toxicity.

## Conclusions

In conclusion, this case highlights the significant risk of methotrexate-induced toxicity in patients with pre-existing chronic kidney disease, particularly when dosing adjustments are not appropriately made. The timely recognition of methotrexate-induced pancytopenia and the administration of intravenous leucovorin were crucial in reversing the hematological complications. The importance of careful medication reconciliation and monitoring in individuals with multiple comorbidities emphasizes the need for a multidisciplinary approach to managing such cases. Pharmacogenomic analysis of methotrexate could enable pre-treatment assessments to predict both efficacy and toxicity risks.
